# New approaches to neglected phenomena

**DOI:** 10.1590/S1980-57642009DN30300001

**Published:** 2009

**Authors:** Ricardo Nitrini

**Affiliations:** Editor-in-Chief

In this issue of **Dementia & Neuropsychologia**, Nunes and colleagues present an interesting paper evaluating the effect of educational level on the phenomenon of reduction of the asymmetry of processing by the cerebral hemispheres with aging. An intriguing observation was that although no difference between individuals with low or high educational level was seen on cognitive tests, differences were in cerebral processing were evident on magnetoencephalography.^1^ This group of researchers led by Alexandre Castro-Caldas, of the University of Lisbon, Portugal, has been investigating the effects of illiteracy and low educational level on the brain by means of more current and sophisticated methods of analysis of the functional organization of the central nervous system.

In 1998, Castro-Caldas and colleagues in their already classic study, employed positron emission tomography (PET) with statistical parametric mapping to evaluate the differences between illiterates and non-illiterates on verbal tasks, and concluded that the functional organization of the adult brain is modified by learning to read and write in childhood. At the time the study was devised, PET was not available in Portugal, so the authors had to seek support from the Karolinska Institute of Stockholm, Sweden, where patients were flown to undergo the examination.^2^ The relevance of taking advantage of more elaborate methods to study hitherto less investigated phenomena, or those have been evaluated using only clinical or conventional research methods, is one of the most important non explicit contributions of this group of researchers and is reflected by the paper published in this issue.

The use of novel methods or models of study has been critical for the advance of science. Indeed, it was the application of the silver impregnation methods, only recently reported by Camilo Golgi at the time, that allowed Santiago Ramón y Cajal to go on and produce what is probably the most seminal page on neurosciences ever written by any one individual.^3^ Within the sphere of particular interest to **Dementia & Neuropsychologia**, it is apt to remind that the modifications of the silver impregnation methods proposed by Max Bielschowsky paved the way for Alois Alzheimer to describe the pathological hallmarks of Alzheimer’s disease,^4^ while it was the very appropriate choice of *Aplysia californica* that made possible many of the remarkable advances in mechanisms of memory achieved by Eric Kandel and his team.^5^

Numbering among the main motivations of **Dementia & Neuropsychologia** is the publication of papers investigating the effects of education and cultural phenomena on the central nervous system as well as studies on diseases that are more common in developing countries than in developed regions. Methods are the essence of science and new methods and models will certainly shed new and brighter light on these relatively neglected fields.
